# Performance of and Severe Acute Respiratory Syndrome Coronavirus 2 Diagnostics Based on Symptom Onset and Close Contact Exposure: An Analysis From the Test Us at Home Prospective Cohort Study

**DOI:** 10.1093/ofid/ofae304

**Published:** 2024-05-31

**Authors:** Carly Herbert, Biqi Wang, Honghuang Lin, Yi Yan, Nathaniel Hafer, Caitlin Pretz, Pamela Stamegna, Colton Wright, Thejas Suvarna, Emma Harman, Summer Schrader, Chris Nowak, Vik Kheterpal, Elizabeth Orvek, Steven Wong, Adrian Zai, Bruce Barton, Ben S Gerber, Stephenie C Lemon, Andreas Filippaios, Laura Gibson, Sharone Greene, Andres Colubri, Chad Achenbach, Robert Murphy, William Heetderks, Yukari C Manabe, Laurel O’Connor, Nisha Fahey, Katherine Luzuriaga, John Broach, Kristian Roth, David D McManus, Apurv Soni

**Affiliations:** Program in Digital Medicine, Department of Medicine, University of Massachusetts Chan Medical School, Worcester, Massachusetts, USA; University of Massachusetts Center for Clinical and Translational Science, University of Massachusetts Chan Medical School, Worcester, Massachusetts, USA; Program in Digital Medicine, Department of Medicine, University of Massachusetts Chan Medical School, Worcester, Massachusetts, USA; Division of Health System Science, Department of Medicine, University of Massachusetts Chan Medical School, Worcester, Massachusetts, USA; Program in Digital Medicine, Department of Medicine, University of Massachusetts Chan Medical School, Worcester, Massachusetts, USA; Division of Health System Science, Department of Medicine, University of Massachusetts Chan Medical School, Worcester, Massachusetts, USA; Division of Microbiology, OHT7 Office of Product Evaluation and Quality, Center for Devices and Radiological Health, US Food and Drug Administration, Silver Spring, Maryland, USA; University of Massachusetts Center for Clinical and Translational Science, University of Massachusetts Chan Medical School, Worcester, Massachusetts, USA; Program in Molecular Medicine, University of Massachusetts Chan Medical School, Worcester, Massachusetts, USA; Program in Digital Medicine, Department of Medicine, University of Massachusetts Chan Medical School, Worcester, Massachusetts, USA; Program in Digital Medicine, Department of Medicine, University of Massachusetts Chan Medical School, Worcester, Massachusetts, USA; Program in Digital Medicine, Department of Medicine, University of Massachusetts Chan Medical School, Worcester, Massachusetts, USA; CareEvolution, Ann Arbor, Michigan, USA; CareEvolution, Ann Arbor, Michigan, USA; CareEvolution, Ann Arbor, Michigan, USA; CareEvolution, Ann Arbor, Michigan, USA; CareEvolution, Ann Arbor, Michigan, USA; Department of Population and Quantitative Health Sciences, University of Massachusetts Chan Medical School, Worcester, Massachusetts, USA; Department of Population and Quantitative Health Sciences, University of Massachusetts Chan Medical School, Worcester, Massachusetts, USA; Department of Population and Quantitative Health Sciences, University of Massachusetts Chan Medical School, Worcester, Massachusetts, USA; Department of Population and Quantitative Health Sciences, University of Massachusetts Chan Medical School, Worcester, Massachusetts, USA; Department of Population and Quantitative Health Sciences, University of Massachusetts Chan Medical School, Worcester, Massachusetts, USA; Department of Population and Quantitative Health Sciences, University of Massachusetts Chan Medical School, Worcester, Massachusetts, USA; Program in Digital Medicine, Department of Medicine, University of Massachusetts Chan Medical School, Worcester, Massachusetts, USA; Division of Infectious Disease, Department of Medicine, University of Massachusetts Chan Medical School, Worcester, Massachusetts, USA; Division of Infectious Disease, Department of Medicine, University of Massachusetts Chan Medical School, Worcester, Massachusetts, USA; Department of Microbiology and Physiological Systems, University of Massachusetts Chan Medical School, Worcester, Massachusetts, USA; Division of Infectious Disease, Department of Medicine, Havey Institute for Global Health, Feinberg School of Medicine, Northwestern University, Chicago, Illinois, USA; Division of Infectious Disease, Department of Medicine, Havey Institute for Global Health, Feinberg School of Medicine, Northwestern University, Chicago, Illinois, USA; National Institute of Biomedical Imaging and Bioengineering, NIH, via contract with Kelly Services, Bethesda, Maryland, USA; Division of Infectious Disease, Department of Medicine, Johns Hopkins University School of Medicine, Baltimore, Maryland, USA; Department of Emergency Medicine, University of Massachusetts Chan Medical School, Worcester, Massachusetts, USA; Program in Digital Medicine, Department of Medicine, University of Massachusetts Chan Medical School, Worcester, Massachusetts, USA; Department of Population and Quantitative Health Sciences, University of Massachusetts Chan Medical School, Worcester, Massachusetts, USA; Department of Pediatrics, University of Massachusetts Chan Medical School, Worcester, Massachusetts, USA; University of Massachusetts Center for Clinical and Translational Science, University of Massachusetts Chan Medical School, Worcester, Massachusetts, USA; Program in Molecular Medicine, University of Massachusetts Chan Medical School, Worcester, Massachusetts, USA; University of Massachusetts Center for Clinical and Translational Science, University of Massachusetts Chan Medical School, Worcester, Massachusetts, USA; Department of Emergency Medicine, University of Massachusetts Chan Medical School, Worcester, Massachusetts, USA; Division of Microbiology, OHT7 Office of Product Evaluation and Quality, Center for Devices and Radiological Health, US Food and Drug Administration, Silver Spring, Maryland, USA; Program in Digital Medicine, Department of Medicine, University of Massachusetts Chan Medical School, Worcester, Massachusetts, USA; Division of Health System Science, Department of Medicine, University of Massachusetts Chan Medical School, Worcester, Massachusetts, USA; Division of Cardiology, Department of Medicine, University of Massachusetts Chan Medical School, Worcester, Massachusetts, USA; Program in Digital Medicine, Department of Medicine, University of Massachusetts Chan Medical School, Worcester, Massachusetts, USA; Division of Health System Science, Department of Medicine, University of Massachusetts Chan Medical School, Worcester, Massachusetts, USA; Department of Population and Quantitative Health Sciences, University of Massachusetts Chan Medical School, Worcester, Massachusetts, USA

**Keywords:** SARS-CoV-2, rapid antigen test, SARS-CoV-2 variant, COVID-19, RT-PCR

## Abstract

**Background:**

Understanding changes in diagnostic performance after symptom onset and severe acute respiratory syndrome coronavirus 2 (SARS-CoV-2) exposure within different populations is crucial to guide the use of diagnostics for SARS-CoV-2.

**Methods:**

The Test Us at Home study was a longitudinal cohort study that enrolled individuals across the United States between October 2021 and February 2022. Participants performed paired antigen-detection rapid diagnostic tests (Ag-RDTs) and reverse-transcriptase polymerase chain reaction (RT-PCR) tests at home every 48 hours for 15 days and self-reported symptoms and known coronavirus disease 2019 exposures immediately before testing. The percent positivity for Ag-RDTs and RT-PCR tests was calculated each day after symptom onset and exposure and stratified by vaccination status, variant, age category, and sex.

**Results:**

The highest percent positivity occurred 2 days after symptom onset (RT-PCR, 91.2%; Ag-RDT, 71.1%) and 6 days after exposure (RT-PCR, 91.8%; Ag-RDT, 86.2%). RT-PCR and Ag-RDT performance did not differ by vaccination status, variant, age category, or sex. The percent positivity for Ag-RDTs was lower among exposed, asymptomatic than among symptomatic individuals (37.5% (95% confidence interval [CI], 13.7%–69.4%) vs 90.3% (75.1%–96.7%). Cumulatively, Ag-RDTs detected 84.9% (95% CI, 78.2%–89.8%) of infections within 4 days of symptom onset. For exposed participants, Ag-RDTs detected 94.0% (95% CI, 86.7%–97.4%) of RT-PCR–confirmed infections within 6 days of exposure.

**Conclusions:**

The percent positivity for Ag-RDTs and RT-PCR tests was highest 2 days after symptom onset and 6 days after exposure, and performance increased with serial testing. The percent positivity of Ag-RDTs was lowest among asymptomatic individuals but did not differ by sex, variant, vaccination status, or age category.

## Teaser

Rapid antigen tests had highest diagnostic performance 2 days after symptom onset and 6 days after exposure to severe acute respiratory syndrome coronavirus 2 (SARS-CoV-2). Diagnostic performance did not differ by vaccination status, age, sex, or SARS-CoV-2 variant.

Antigen-detection rapid diagnostic tests (Ag-RDTs) are commonly used to diagnose coronavirus disease 2019 (COVID-19) due to their availability for home use, relatively low cost, and ability to return results in 15–20 minutes [1, 2]. Previous US Food and Drug Administration Safety Communications describe methods to minimize the risk of false-negative COVID-19 antigen test results in symptomatic and asymptomatic individuals [3, 4]. However, important questions remain about when to begin testing, particularly among those with symptoms or after close contact with an infected person [5, 6].

Many demographic and viral factors, including age, sex, vaccination status, and severe acute respiratory syndrome coronavirus (SARS-CoV-2) variant, have been associated with changes and differences in symptoms and viral kinetics of SARS-CoV-2 infection [7]. Vaccination, younger age, and infection with the Omicron variant have been associated with fewer symptoms, lower severity of infection, and a higher likelihood of asymptomatic infections [8–10]. The incubation period, or the time from exposure to infection, has been found to differ by variant and age category, which informs testing strategies after exposure to SARS-CoV-2 [11–14]. Furthermore, men have been found to have higher mean and peak viral loads than women throughout SARS-CoV-2 infection [15]. The performance of molecular diagnostics, including reverse-transcriptase polymerase chain reaction (RT-PCR) tests and Ag-RDTs is closely related to detectable viral load; therefore, it is important to determine whether these differences in viral dynamics and symptoms have an impact on diagnostic performance [3].

Using data from the prospective cohort study, Test Us at Home [16], we examined home-collected paired serial Ag-RDTs and RT-PCR tests to compare the percent positivity of these tests and how they differed by time since symptom onset and exposure. We also explored how these findings varied based on vaccination status, variant, sex, and age. The results of this study will inform pragmatic use of at-home Ag-RDTs to detect SARS-CoV-2.

## METHODS

### Study Population

We used data from the Test Us at Home study, a longitudinal cohort study that evaluated the performance of serial use of Ag-RDTs for detection of COVID-19 [16]. This study enrolled participants aged ≥2 years across the United States between October 2021 and February 2022. The Test Us at Home study aimed to understand the performance of SARS-CoV-2 diagnostics during the onset of infection and in asymptomatic infections. Participants were required to be asymptomatic on enrollment, and recruitment was targeted toward communities across the continental United States with a high incidence of SARS-CoV-2 infection. All participants provided written consent for this study, which was approved by the WIRB-Copernicus Group Institutional Review Board (no. 20214875).

All Test Us at Home participants were asked to conduct Ag-RDT and RT-PCR testing every 48 hours over a 15-day period and record their results within a study app. Every 48 hours, participants received a push notification in the study app, which notified them to begin testing. Additional reminders were sent every 2 hours until testing was complete. During each testing session, 2 anterior nasal swab samples were self-collected at home; one swab was used for performing an Ag-RDT, while the other was sent to a central laboratory for RT-PCR testing. Additional detail about the study design, protocol, and participants are described elsewhere [3, 16].

Only participants who completed ≥1 Ag-RDT or RT-PCR test were included in this analysis. Participants were included in the day past symptom onset (DPSO) analyses if they self-reported any symptoms during the study period and had ≥1 positive RT-PCR result (Supplementary Figure 1). Participants who had an RT-PCR–positive result >14 days before or after symptom onset were excluded, as these symptoms were assumed to be unrelated to the observed infection [17]. Furthermore, participants who reported symptoms on the first day of testing were excluded, to ensure that we most accurately calculated DPSO 0. Participants who reported exposure to SARS-CoV-2 before their RT-PCR–positive result were included in the day past exposure (DPE) analysis. Those with an index RT-PCR–positive result >14 days after the reported exposure were excluded from the DPE analyses. Participants with both symptoms and exposure were included in both analyses if they met both eligibility criteria.

### Measures

Participants were prompted to self-report symptoms (fever, body aches, fatigue, rash, nausea, abdominal pain, diarrhea, loss of smell, runny nose, cough, headache, or other) every 48 hours, immediately before testing. Participants also had the ability to report the onset of symptoms at any time if symptoms developed between testing periods. The first day that a participant reported ≥1 symptoms was termed DPSO 0.

Participants self-reported close-contact exposures to COVID-19 at the time of baseline study enrollment and before each testing period. They were asked to report the date of their exposure, the proximity of contact, and the duration of exposure with the infected person. An exposure was defined as being within 6 feet of an infected person without a mask for ≥15 minutes over a 24-hour period. DPE 0 was defined as the first day of the reported exposure.

Vaccination status, sex, and age were self-reported during the enrollment survey. Vaccination status was operationalized into 2 groups: vaccinated (≥1 dose) and unvaccinated (0 doses). Self-reported age was used to assign people by age group, as children (<18 years old) or adults (≥18 years old).

For molecular testing (RT-PCR), 2 high-sensitivity RT-PCR assays (Roche Cobas 6800 SARS-CoV-2 PCR and Quest RC COVID-19 PCR DTC) were performed on each anterior nasal swab sample received at Quest Laboratories, and an additional tiebreaker assay (Hologic Aptima SARS-CoV-2 Transcription Mediated Amplification assay) was performed if assay results were discordant. Samples positive on 2 of 3 assays were counted as a true-positive. Cycle threshold (Ct) values for the E gene from Roche Cobas SARS-CoV-2 test were used to quantify viral load. Participants’ SARS-CoV-2 variants were determined using whole-genome sequencing of SARS-CoV-2 by amplicon-based next-generation sequencing on extracted RNA. Participants without sequencing results were excluded in variant stratified analyses (n = 20).

Participants were assigned to 1 of 3 rapid antigen tests: Quidel QuickVue At-Home OTC COVID-19 Test, Abbott BinaxNOW COVID-19 Antigen Self Test, or BD Veritor At-Home COVID-19 Test. Test assignment was determined using an automated algorithm based on enrollment numbers and geographic location of the participants. Participants were provided with test-specific instructions with images, per respective emergency use authorizations, to mimic real-world testing conditions. During each testing session, participants were asked to provide an interpretation of each Ag-RDT result (positive, negative, or invalid) and upload a picture of the test result to the study app. All self-reported positive test results were confirmed by study coordinators using uploaded images.

For data analysis, demographic factors for eligible participants were tabulated and described. Ct values were averaged on each DPSO and DPE, stratified by variant, sex, age category, and vaccination status, and 95% confidence intervals (CIs) were calculated using Wilson's method [18]. Ct values for symptomatic and asymptomatic participants were also calculated by DPE. The percent positivity of symptomatic and/or exposed participants was calculated for RT-PCR tests and Ag-RDTs by DPSO and DPE with 95% CIs [18]. The denominator for percent positivity was the number of participants who tested positive on the composite RT-PCR result at least once during the observation period (DPSO −14 to 14 and DPE 0–14) and recorded a test on that specific DPSO or DPE. The numerator for percent positivity was the number of participants with a positive test result (Ag-RDT or RT-PCR) on each DPSO or DPE.

Cumulative positivity was calculated at DPSO 4, DPSO 6, DPE 4, and DPE 6. This was defined as the sum of participants with ≥1 positive test result between the beginning of the observation period (ie, DPSO −14 or DPE 0) to the day of calculation, divided by the total number participants who tested positive by RT-PCR comparator at least once during the study. In other words, for cumulative positivity on DPSO 4, it would include the participants who tested positive anytime between DPSO −14 to DPSO 4, divided by the total number of participants who tested positive by molecular comparator during the study period. Analyses for DPSO were stratified by vaccination status, variant, sex, and age category. DPE analyses were stratified by vaccination status and symptom status, due to sample size limitations (comparator group n < 20). All analyses were conducted using R software, version 4.2.1 [19].

## RESULTS

### Characteristics of Symptomatic and Exposed Participants

Among the 7361 Test Us At Home participants, 146 tested positive for SARS-CoV-2 during the study period, reported symptoms during their infection, and were asymptomatic at the first test, making them eligible for the DPSO analysis. In addition, 96 participants tested positive after a close-contact exposure to SARS-CoV-2 and were eligible for the DPE analysis (Supplementary Figure 1). The majority of exposed participants (69.8%) reported that their close-contact exposure occurred with someone in their household. Among the participants included in DPE analyses, 85 (88.5%) developed symptoms at some point during their infection and were included in both analyses; the mean time from exposure to symptom onset was 4.69 days (interquartile range, 2–6 days) (Table 1). Most participants (88.4%) were previously uninfected with SARS-CoV-2, between the ages of 18 and 44 years, and female (64.4%). Approximately 60% of participants had received ≥2 doses of a SARS-CoV-2 vaccine, and >30% were unvaccinated. The majority of participants included in DPSO analyses (69.2%) were infected with the Omicron variant.

**Table 1.** Characteristics of Participants Included in Analyses

Characteristic	Participants, No. (%)	
	Included in DPSO (n = 146)	Included in DPE (n = 96)
Symptomatic	146 (100.0)	85 (88.5)
No. of previous infections		
0	129 (88.4)	85 (88.5)
1	14 (9.6)	9 (9.4)
≥2	3 (2.1)	2 (2.1)
Age		
<18 y	22 (15.1)	17 (17.7)
18–44 y	91 (62.3)	52 (54.2)
45–64 y	29 (19.9)	24 (25.0)
≥65 y	4 (2.7)	3 (3.1)
Sex		
Male	47 (32.2)	37 (38.5)
Female	94 (64.4)	58 (60.4)
Missing	5 (3.4)	1 (1.0)
Race		
White	116 (79.5)	81 (84.4)
Asian	7 (4.8)	3 (3.1)
Black/African-American	8 (5.5)	4 (4.2)
Multiracial	8 (5.5)	3 (3.1)
Other	4 (2.8)	5 (5.2)
Missing	3 (2.1)	0 (0.0)
Hispanic	18 (12.3)	6 (6.2)
Educational level		
Bachelor's degree or higher	65 (44.5)	47 (49.0)
Some college	31 (21.2)	18 (18.8)
High school graduate	23 (15.8)	15 (15.6)
Did not finish high school	22 (15.1)	13 (13.5)
Don’t know	0 (0.0)	3 (3.1)
Missing	5 (3.4)	0 (0.0)
Vaccination status		
Unvaccinated	47 (32.2)	32 (33.3)
1 vaccine dose	6 (4.1)	3 (3.1)
≥2 vaccine doses	93 (63.7)	61 (63.5)
SARS-CoV-2 variant		
Delta	34 (23.3)	15 (15.6)
Omicron	101 (69.2)	75 (78.1)
Unknown	11 (7.5)	6 (6.2)

Abbreviation: DPE, day past exposure; DPSO, day past symptom onset; SARS-CoV-2, severe acute respiratory syndrome coronavirus 2.

### Ct Values by DPSO

**Figure 1.**
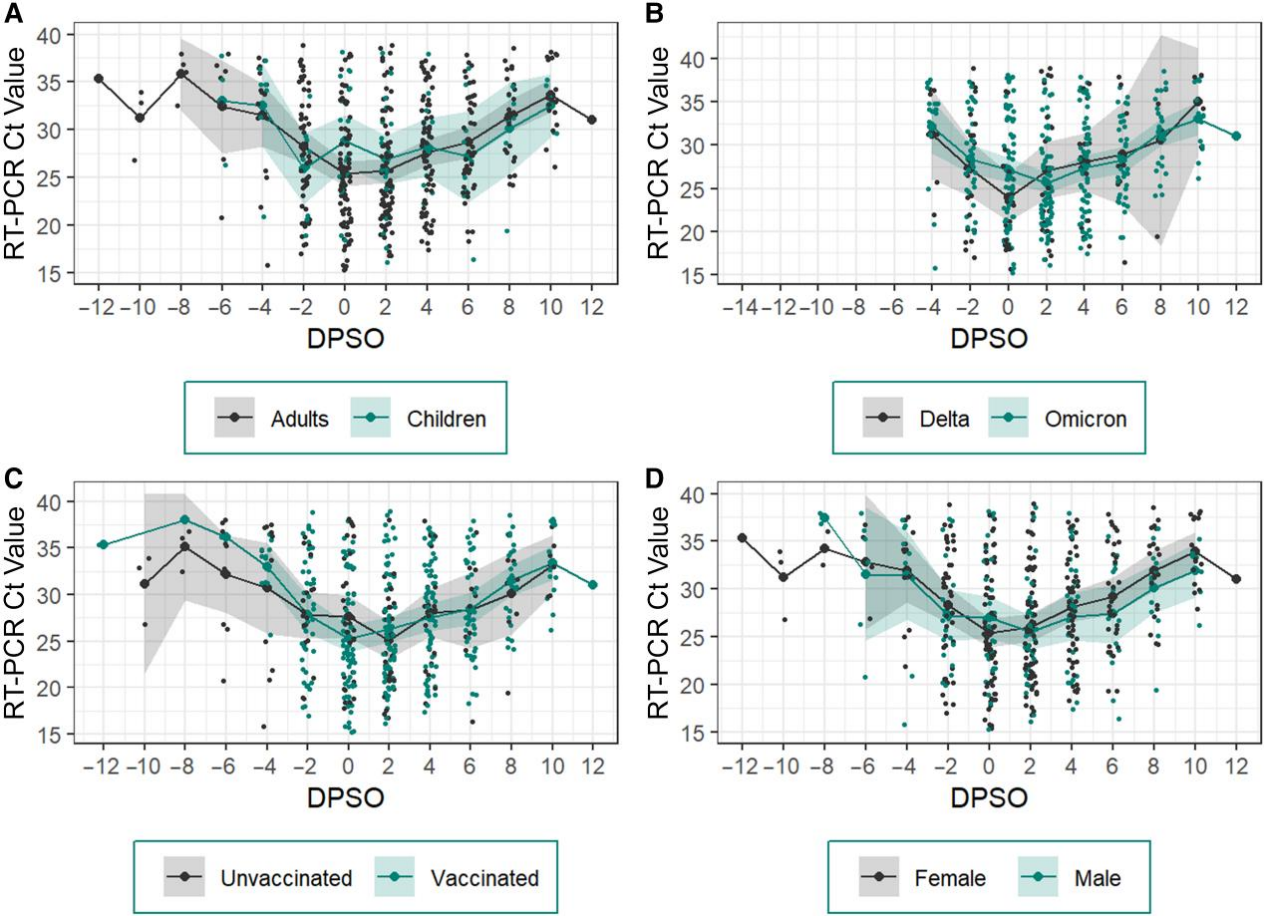
Reverse-transcriptase polymerase chain reaction (RT-PCR) cycle threshold (Ct) values by day past symptom onset (DPSO). Mean Ct values on DPSO among adults and children (*A*), Delta and Omicron and severe acute respiratory syndrome coronavirus 2 variants (*B*), vaccinated and unvaccinated participants (*C*), and female and male participants (*D*). Dots represent individual participant values; lines, mean Ct values; and shaded regions, 95% confidence intervals.

For all symptomatic participants, the mean nadir Ct value was observed on DPSO 2 (Ct, 26.0) (Figure 1). The nadir Ct value for adults occurred on the day of symptom onset (DPSO 0), and Ct values among children were higher than in adults (adults, 25.41 [95% CI, 24.1–26.8]; children, 28.78 [26.0–31.5]) (Figure 1*A*). Participants infected with the Delta variant appeared to experience the nadir Ct value earlier than those infected with the Omicron variant. With the Delta variant, this peak occurred at DPSO 0, or the first day of symptom onset; however, among participants with the Omicron variant, peak viral load occurred on DPSO 2 (Figure 1*B*). Vaccinated participants also experienced their peak viral load earlier than unvaccinated participants (DPSO 0 vs 2) (Figure 1*C*). After DPSO 2, male participants had lower Ct values than female participants, though this difference was not statistically significant (Figure 1*D*). No significant differences in the magnitude of viral loads were observed by DPSO onset among children compared with adults, or by variant, vaccination status, or sex.

### Performance of SARS-CoV-2 Diagnostics by DPSO

**Figure 2.**
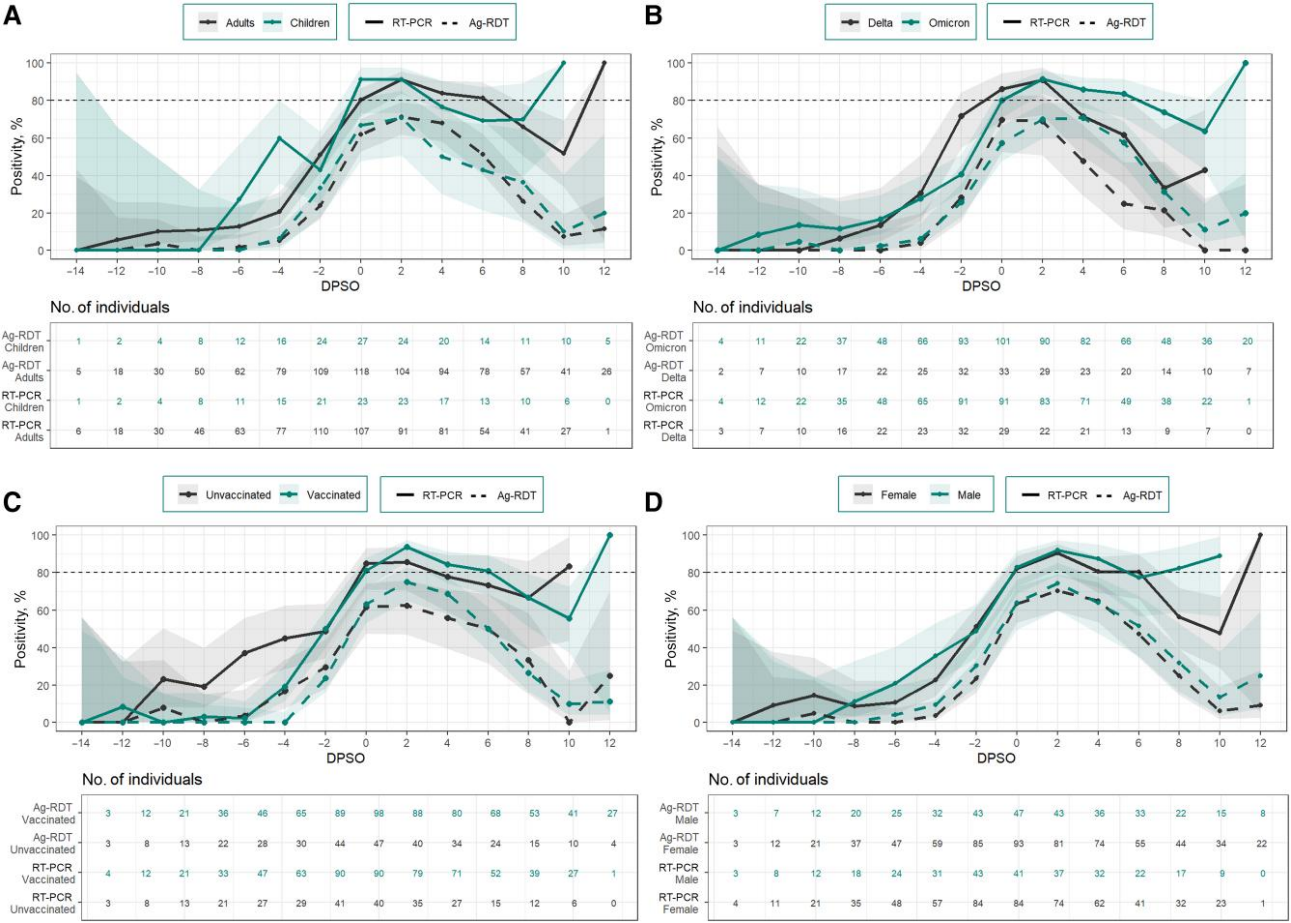
Reverse-transcriptase polymerase chain reaction (RT-PCR) and antigen-detection rapid diagnostic test (Ag-RDT) positivity by day past symptom onset (DPSO). Filled lines represent RT-PCR results; dashed lines, Ag-RDT results; and shaded regions, 95% confidence intervals. Percent positivity of Ag-RDT and RT-PCR by DPSO is shown among adults (*black*) and children (*green*) (*A*), individuals with the Delta (*black*) and Omicron (*green*) variants (*B*), unvaccinated (*black*) and vaccinated (*green*) individuals (*C*), and female (*black*) and male (*green*) participants (*D*). Percent positivity was defined as the number of participants with a positive result (Ag-RDT or RT-PCR) on each DPSO divided by the number of participants who tested positive on RT-PCR at least once during the observation period (DPSO −14 to 14) and recorded a test on that specific DPSO.

Among all symptomatic individuals, RT-PCR tests and Ag-RDTs performed highest on DPSO 2, or 2 days after symptom onset (RT-PCR, 91.2% [95% CI, 84.6%–95.2%]; Ag-RDT, 71.1% [62.7%–78.2%]) (Supplementary Table 1). On DPSO 2, RT-PCR tests detected 91.2% of infections (95% CI, 84.6%–95.2%), and Ag-RDTs detected 71.1% (62.7%–78.2%). RT-PCR testing and Ag-RDTs performed similarly among children and adults (Figure 2*A* and Supplementary Table 2), vaccinated and unvaccinated individuals (Figure 2*C* and Supplementary Table 4), and male and female participants (Figure 2*D* and Supplementary Table 5). Ag-RDTs detected 84.9% of all infections cumulatively by DPSO 4.

On the day of symptom onset (DPSO 0), RT-PCR had a percent positivity of 86.2% (95% CI, 69.4%–94.5%) and 80.2% (70.9%–87.1%) for Delta and Omicron variants, respectively (Figure 2*B* and Supplementary Table 3). For both variants, the highest percent positivity for RT-PCR was achieved on DPSO 2 (Delta, 90.9% [95% CI, 72.2%–97.5%]; Omicron, 91.6% [83.6%–95.9%]). There were no significant differences in the performance of Ag-RDTs between participants with Delta and Omicron variants; however, we did observe that participants with the Omicron variant had their highest percent positivity on DPSO 4 (70.7% [95% CI, 60.1%–79.5%), while those with the Delta variant which had their highest percent positivity on DPSO 0 (69.7% [52.7%–82.6%]).

### Ct Values by DPE

**Figure 3.**
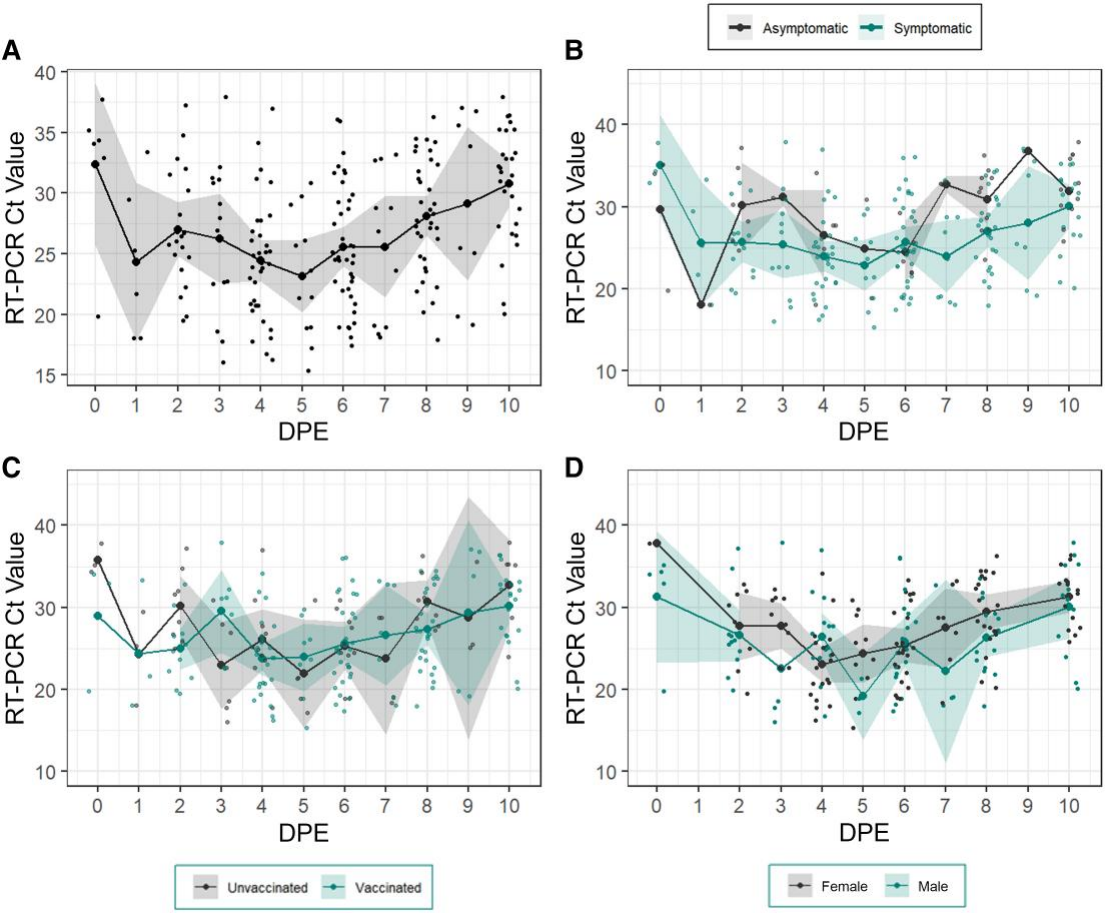
Cycle threshold (Ct) values by day past exposure (DPE). Mean Ct values on day past exposure (DPE) among all exposed participants (*A*), symptomatic and asymptomatic participants (*B*), vaccinated and unvaccinated participants (*C*), and female and male participants (*D*). Dots represent individual participant values; lines, mean Ct values; and shaded regions, 95% confidence intervals.

For participants who experienced a close-contact SARS-CoV-2 exposure, the lowest mean Ct value occurred on DPE 5 (Ct, 23.6 [95% CI, 20.6–26.6]) (Figure 3*A*). Despite wide CIs, symptomatic individuals had lower mean Ct values than asymptomatic individuals throughout the infection period, except on DPE 6, when Ct values were roughly equivalent (Figure 3*B*). We did not observe any differences in Ct values between vaccinated and unvaccinated participants (Figure 3*C*). Finally, male participants appeared to have lower Ct values than female participants throughout their infections; however, due to sample size, CIs were wide (Figure 3*D*).

### Performance of SARS-CoV-2 Diagnostics by DPE

**Figure 4.**
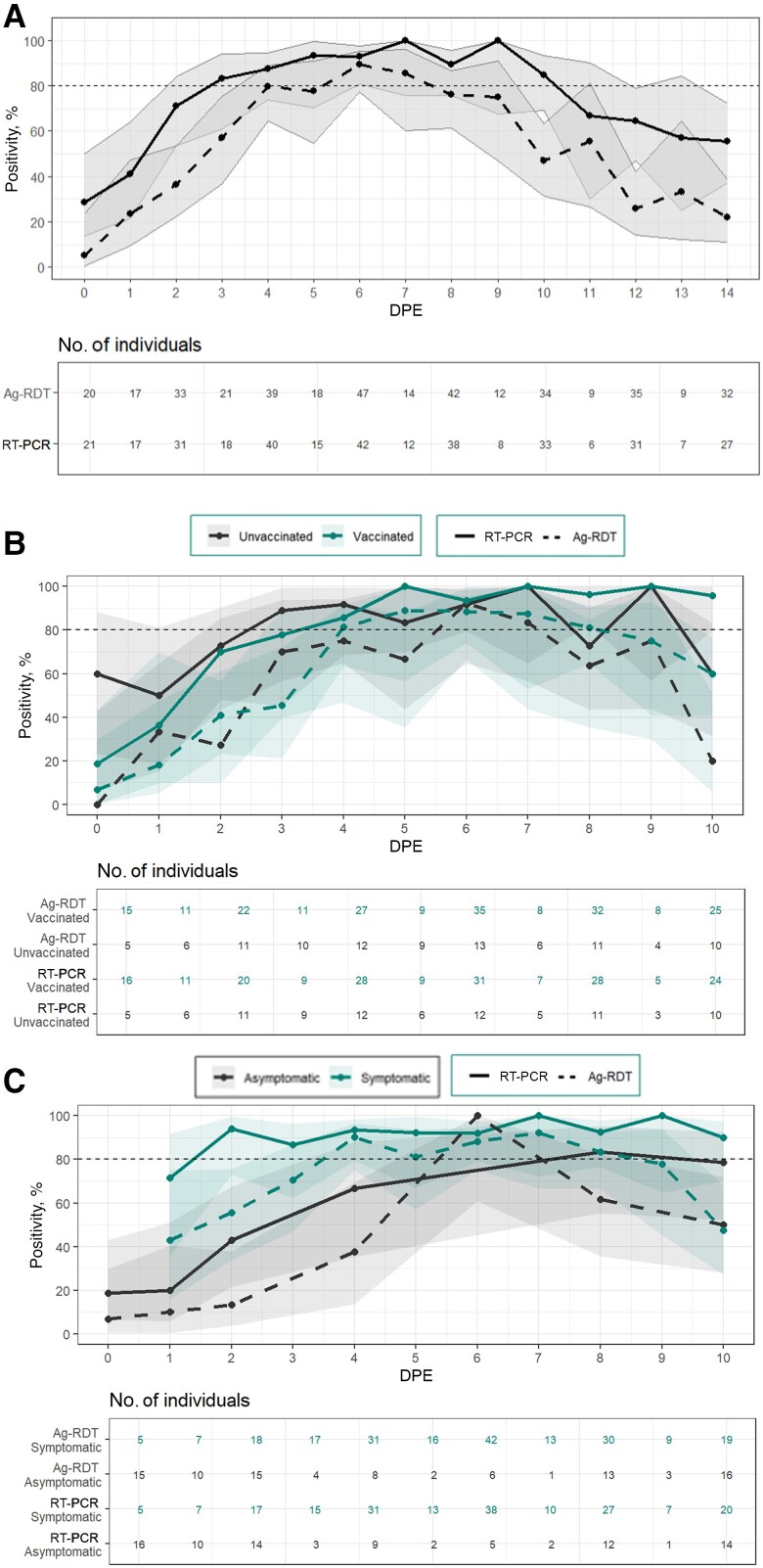
Reverse-transcriptase polymerase chain reaction (RT-PCR) and antigen-detection rapid diagnostic test (Ag-RDT) positivity by day past exposure (DPE). Filled lines represent RT-PCR results; dashed lines, Ag-RDT results; shaded regions, 95% confidence intervals. Exposure was defined as being within 6 feet of an infected person without a mask for ≥15 minutes over a 24-hour period. DPE 0 was defined as the first day of the reported exposure. The percent positivity for Ag-RDTs and RT-PCR tests by day past exposure is shown among all exposed participants (*A*), unvaccinated (*black*) and vaccinated individuals (*green*) (*B*), and asymptomatic (*black*) and symptomatic (*green*) participants (*C*). Percent positivity was defined as the number of participants with a positive test result (Ag-RDT or RT-PCR) on each DPE divided by the number who tested positive with RT-PCR at least once during the observation period (DPE 0 to 10) and recorded a test on that specific DPE.

Among exposed individuals, the percent positivity for RT-PCR tests and Ag-RDTs was highest on DPE 6, or 6 days after close-contact exposure to SARS-CoV-2 (Figure 4*A* and Supplementary Table 6). On DPE 6, RT-PCR detected 91.8% of infections (95% CI, 80.8%–96.8%), and Ag-RDTs detected 86.2% (75.1%–92.8%). Among vaccinated individuals, the highest percent positivity of RT-PCR was on DPE 8, with percent positivity of 96.6% (95% CI, 82.8%–99.8%) (Figure 4*B*). For unvaccinated participants, the highest percent positivity of RT-PCR occurred on DPE 6 (93.8% [95% CI, 71.7%–99.7%]). The highest percent positivity for Ag-RDTs occurred on DPE 6 among both vaccinated and unvaccinated individuals. We observed no differences between percent positivity for RT-PCR and Ag-RDTs between male and female participants.

The percent positivity of RT-PCR for exposed, symptomatic participants was consistently high (>90%) starting at DPE 2 (Figure 4*C* and Supplementary Table 7). The percent positivity for Ag-RDTs among symptomatic, exposed participants peaked at DPE 4 (90.3% [95% CI, 75.1%–96.7%). Though CIs were wide, the performances of Ag-RDTs and RT-PCR were consistently lower among asymptomatic individuals, and the percent positivity on DPE 4 was 66.7% (95% CI, 35.4%–87.9%) for RT-PCR and 37.5% (13.7%–69.4%) for Ag-RDTs.

## DISCUSSION

We report the performance of nasal-swab Ag-RDTs and RT-PCR in home-based settings by time since symptom onset and exposure by sex, age category, vaccination status, and variant. We identified 3 important findings: (1) the performance of Ag-RDTs and RT-PCR peaked on DPSO 2 for symptomatic individuals and on DPE 6 for exposed individuals; (2) the timing of viral peak did not differ by sex, vaccination status, or age; (3) the performance of SARS-CoV-2 diagnostic tests is similar among vaccinated and unvaccinated participants, adults and children, and male and female participants and for Delta and Omicron variants. Taken together, these findings reinforce the importance of Ag-RDTs for detection of SARS-CoV-2 virus.

As the pandemic enters its fourth year, use of COVID-19 diagnostics has shifted away from general screening and mandated testing toward personal risk assessment, with most people using Ag-RDTs in response to acute symptoms or COVID-19 exposure [20]. It is increasingly important to advise individuals on the timing of Ag-RDT use, to facilitate accurate test interpretation and minimize false-negative results. The present results reinforce the importance of serial testing when individuals are either symptomatic or exposed to SARS-CoV-2, in line with previous recommendations [4]. For symptomatic individuals in our study, >85% of PCR-confirmed infections were detected with Ag-RDTs by DPSO 4, indicating that serial testing on DPSO 2 and DPSO 4 offers an effective strategy for detection of SARS-CoV-2 with Ag-RDTs. Among exposed participants, Ag-RDTs detected nearly 80% of PCR-confirmed infections cumulatively by DPE 6, and performance was highest on DPE 6. We observed lower performance of Ag-RDTs among asymptomatic, exposed individuals compared with symptomatic, exposed individuals, also indicating the need for serial testing within this group.

Several prior studies have examined Ag-RDT performance when tests are used serially, but these studies predate the arrival of the Omicron variants in the United States and widespread vaccination coverage [21, 22]. We observed that viral load peaked on DPSO 4 for participants with the Omicron variant, compared with DPSO 0 for the Delta variant, consistent with other recent studies [23]. However, we did not observe that Ag-RDT performance differed significantly by variant, despite the differences in viral peaks.

A previous study of 225 individuals with PCR-confirmed SARS-CoV-2 infections in the spring of 2021 similarly found that RT-PCR had a positivity rate of approximately 60% on the day of illness onset (defined as symptom onset among symptomatic individuals and first RT-PCR–positive result among asymptomatic individuals) [22]. These investigators also found that Ag-RDTs had lower sensitivity among participants with ≥1 dose of the SARS-CoV-2 vaccine, which differs from our own findings. The observed difference may be explained by the vaccine's declining effect on immunity over time, as many individuals in our study had received the SARS-CoV-2 vaccine >6 months before enrollment, as well as the difference in SARS-CoV-2 variants.

The SARS-CoV-2 vaccine has been found to have lower efficacy in preventing infection from the Delta and Omicron variants compared with previous variants, and studies have found no effect of the SARS-CoV-2 vaccine on symptomatic disease 20 weeks after vaccination [24, 25]. We also observed that peak viral load did not differ between vaccinated and unvaccinated individuals, consistent with previous reports [26]. This further emphasizes the value of reevaluating SARS-CoV-2 diagnostic performance as new variants continue to arise.

Children have consistently demonstrated milder clinical presentations than adults when infected with SARS-CoV-2; however, the mechanism behind this difference between children and adults remains unknown [27, 28]. Pediatric patients often present with fewer or atypical symptoms than adults, which can complicate diagnosis [29]. Furthermore, most previous research on pediatric SARS-CoV-2 infections has occurred in hospitalized patients, even though the majority of pediatric infections are mild and self-limited. Despite the differences in severity of infections among children and adults, our results match previous findings, which showed no differences in viral loads between children and adults [30, 31]. Although viral load did not differ significantly between children and adults, it has been suggested that the difference in severity among children and adults may be associated with a faster rate of viral clearance, leading to milder infections [32]. Also notably, diagnostic performance did not differ significantly between the 2 groups.

Finally, previous studies have found that males have higher peak viral loads than females during infection after adjusting for symptoms, as well as higher rates of severe infection and mortality rates [15, 33]. We did not observe differences in diagnostic performance by sex, nor did we see substantial differences in viral load by DPSO. This may be due to sociological differences in how men and women report their symptoms and perceived risk [34–36], as well as the potential immunological differences in the timing of symptom onset within an infection among men and women, which were not addressed in the design of the current study [37, 38]. Future studies examining objective symptom measures, including temperature, may provide additional information in assessing the comparison of diagnostic performance between men and women by DPSO.

Among the strengths of the current study, it is one of the first to analyze the longitudinal diagnostic performance of RT-PCR tests and Ag-RDTs for COVID-19 based on days past acute symptom onset or exposure to SARS-CoV-2. It assessed serial paired longitudinal data to evaluate the performance of Ag-RDTs and RT-PCR over the duration of infection, using a large nationwide sample of children and adults. It is also, to the best of our knowledge, the first study to quantify time from exposure to Ag-RDT positivity.

However, our study also has limitations. Paired Ag-RDT and RT-PCR testing, as well as symptom trackers, were completed by participants every 48 hours, but participants were able to report symptoms outside these windows. Assessing diagnostic performance at a finer temporal resolution may be useful in future studies. Symptoms, exposures, and Ag-RDT results were based on participant self-report. In this analysis, we grouped anyone with ≥1 vaccine for SARS-CoV-2 as vaccinated due to sample size limitations; however, there may be heterogeneity in the vaccine responses and immunity within this group. In addition, data collection for this study occurred between October 2021 and February 2022, which captured the transition from the predominance of the Delta to the Omicron variant; however, SARS-CoV-2 has continued to evolve, and current circulating Omicron subvariants may have significant differences in transmission and viral dynamics compared to previous strains [39, 40]. Additional research is indicated to examine diagnostic differences in current and future circulating strains. Finally, due to sample size limitations, we were unable to analyze performance by DPE by sex, age, and variant.

In conclusion, the percent positivity for Ag-RDTs and RT-PCR tests was highest on DPSO 2 and DPE 6, and performance increased with serial testing. The performance of Ag-RDTs was lowest among asymptomatic individuals but did not differ by sex, variant, vaccination status, or age category. This confirms that Ag-RDTs remain a useful tool across populations for detection of SARS-CoV-2 in the setting of symptoms or exposures.

## Supplementary Material

ofae304_Supplementary_Data

## Supplementary Data

Supplementary materials are available at *Open Forum Infectious Diseases* online. Consisting of data provided by the authors to benefit the reader, the posted materials are not copyedited and are the sole responsibility of the authors, so questions or comments should be addressed to the corresponding author.
