# Supplementary material for: Performance of and Severe Acute Respiratory Syndrome Coronavirus 2 Diagnostics Based on Symptom Onset and Close Contact Exposure: An Analysis From the Test Us at Home Prospective Cohort Study
Source: Open Forum Infect Dis. 2024 May 31;11(6):ofae304. doi: 10.1093/ofid/ofae304 (PMC11191649; doi:10.1093/ofid/ofae304)
Supplement: ofae304_Supplementary_Data [file ofae304_supplementary_data.pdf]

Supplemental Figure and Tables: Performance of SARS-CoV-2 Diagnostics Based on Symptom Onset and Close Contact Exposure: An analysis from the Test Us At Home prospective cohort study

Contents

Supplemental Figures: 2

Figure S1: Consort Diagram for Day Past Symptom Onset and Day Past Exposure Analyses 2

Supplemental Tables: 3

Table S1: Percent Positivity of Ag-RDT and RT-PCR by Day Past Symptom Onset 3

Table S2: Percent Positivity of Ag-RDT and RT-PCR by Day Past Symptom Onset Among Children and Adults among Symptomatic RT-PCR positive Participants 4

Table S3: Percent Positivity of Ag-RDT and RT-PCR by Day Past Symptom Onset by Variant among Symptomatic RT-PCR Positive Participants 5

Table S4: Percent Positivity of Ag-RDT and RT-PCR by Day Past Symptom Onset by Vaccination Status among Symptomatic RT-PCR Positive Participants 6

Table S5: Percent Positivity of Ag-RDT and RT-PCR by Day Past Symptom Onset Among Males and Females among Symptomatic RT-PCR Positive Participants 7

Table S6: Percent Positivity of Ag-RDT and RT-PCR by Day Past Exposure among Vaccinated and Unvaccinated RT-PCR Positive Participants 8

Table S7: Performance of Ag-RDT and RT-PCR by Day Past Exposure among Symptomatic and Asymptomatic RT-PCR Positive Participants 9

Supplemental Figures:

Figure S1: Consort Diagram for Day Past Symptom Onset and Day Past Exposure Analyses

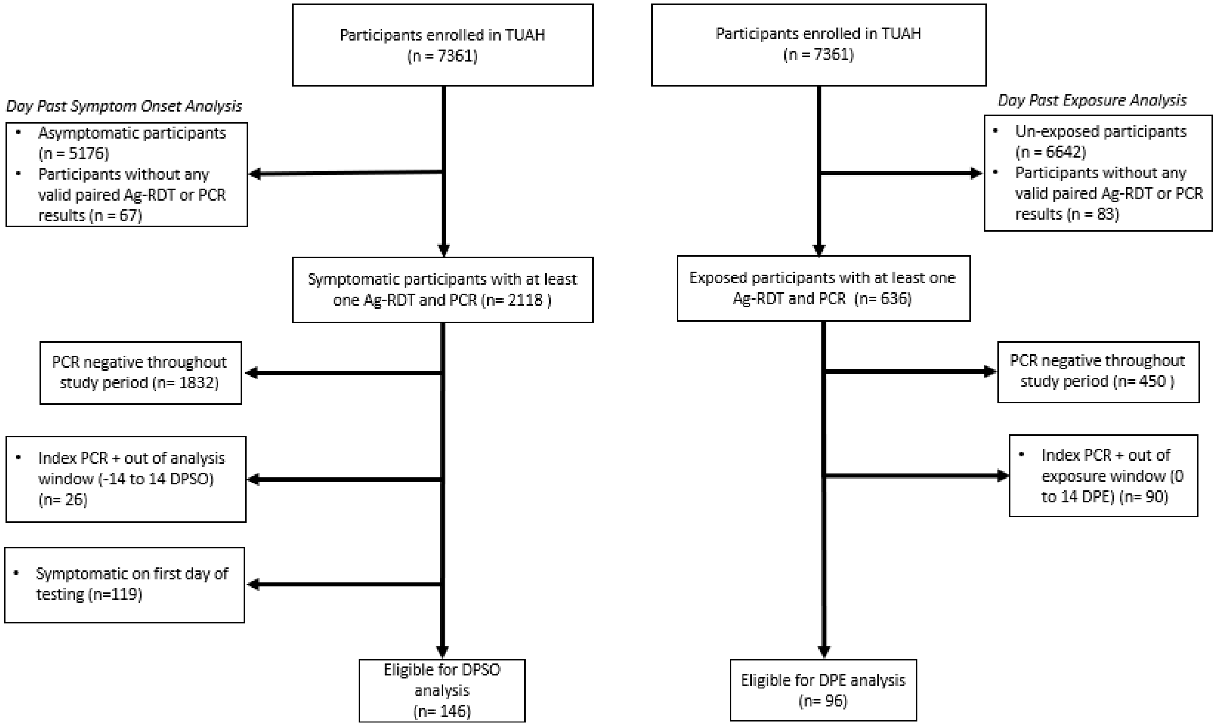

Legend: TUAH: Test Us at Home; PCR: Polymerase chain reaction; Ag-RDT: rapid antigen test; DPSO: Day past symptom onset; DPE: Day past exposure

Supplemental Tables:

Table S1: Percent Positivity of Ag-RDT and RT-PCR by Day Past Symptom Onset

	**All Individuals**	
**Day Past Symptom Onset (DPSO)**	RT-PCR (95% Confidence Interval)	Ag-RDT (95% Confidence Interval)
**-4**	27.17 (19.14-37.04)	5.26 (2.27-11.73)
**-2**	49.62 (41.19-58.07)	25.56 (18.91-33.59)
**0**	82.31 (74.85-87.91)	62.76 (54.66-70.20)
**2**	91.23 (84.60-95.17)	71.09 (62.72-78.24)
**4**	82.65 (73.96-88.88)	64.91 (55.80-73.06)
**6**	79.10 (67.93-87.12)	50.00 (39.99-60.01)
**Cumulative to DPSO 4**	97.95 (94.13-99.30)	84.93 (78.24-89.83)
**Cumulative to DPSO 6**	99.31 (96.22-99.96)	86.99 (80.57-91.51)

Table S2: Percent Positivity of Ag-RDT and RT-PCR by Day Past Symptom Onset Among Children and Adults among Symptomatic RT-PCR positive Participants

	**Children**		**Adults**	
**Day Past Symptom Onset (DPSO)**	RT-PCR (95% Confidence Interval)	Ag-RDT (95% Confidence Interval)	RT-PCR (95% Confidence Interval)	Ag-RDT (95% Confidence Interval)
**-4**	60.00 (35.75-80.18)	6.25 (0.32-28.33)	20.78 (13.22-31.12)	5.06 (1.99-12.31)
**-2**	42.86 (24.47-63.45)	33.33 (17.97-53.29)	50.91 (41.70-60.06)	23.85 (16.83-32.66)
**0**	91.30 (73.20-97.58)	66.67 (47.82-81.36)	80.37 (71.85-86.79)	61.86 (52.86-70.12)
**2**	91.30 (73.20-97.58)	70.83 (50.83-85.09)	91.21 (83.60-95.48)	71.15 (61.82-78.98)
**4**	76.47 (52.74-90.44)	50.00 (29.93-70.07)	83.95 (74.45-90.37)	68.09 (58.11-76.64)
**6**	69.23 (42.37-87.32)	42.86 (21.38-67.41)	81.48 (69.16-89.62)	51.28 (40.39-62.05)
**Cumulative to DPSO 4**	100.00 (87.54-100.00)	85.19 (67.52-94.08)	97.47 (92.85-99.14)	84.87 (77.35-90.21)
**Cumulative to DPSO 6**	100.00 (87.54-100.00)	88.89 (71.94-96.15)	99.16 (95.39-99.96)	86.55 (79.27-91.55)

Table S3: Percent Positivity of Ag-RDT and RT-PCR by Day Past Symptom Onset by Variant among Symptomatic RT-PCR Positive Participants

	**Delta Variant**		**Omicron Variant**	
**Day Past Symptom Onset (DPSO)**	RT-PCR (95% Confidence Interval)	Ag-RDT (95% Confidence Interval)	RT-PCR (95% Confidence Interval)	Ag-RDT (95% Confidence Interval)
**-4**	30.43 (15.60-50.87)	4.00 (0.21-19.54)	27.69 (18.29-39.58)	6.06 (2.38-14.57)
**-2**	71.88 (54.63-84.44)	28.13 (15.56-45.37)	40.66 (31.14-50.93)	25.81 (18.00-35.53)
**0**	86.21 (69.44-94.50)	69.70 (52.66-82.62)	80.22 (70.89-87.11)	57.43 (47.69-66.62)
**2**	90.91 (72.19-97.47)	68.97 (50.77-82.72)	91.57 (83.60-95.85)	70.00 (59.87-78.49)
**4**	71.43 (50.04-86.19)	47.83 (29.24-67.04)	85.92 (75.98-92.17)	70.73 (60.13-79.47)
**6**	61.54 (35.52-82.29)	25.00 (11.19-46.87)	83.67 (70.96-91.49)	57.58 (45.56-68.76)
**Cumulative to DPSO 4**	100.00 (89.85-100.00)	79.41 (63.29-89.65)	98.02 (93.07-99.46)	86.14 (78.07-91.56)
**Cumulative to DPSO 6**	100.00 (89.85-100.00)	82.35 (66.49-91.65)	99.01 (94.60-99.95)	88.12 (80.37-93.07)

Table S4: Percent Positivity of Ag-RDT and RT-PCR by Day Past Symptom Onset by Vaccination Status among Symptomatic RT-PCR Positive Participants

	**Vaccinated Individuals**		**Unvaccinated Individuals**	
**Day Past Symptom Onset (DPSO)**	RT-PCR (95% Confidence Interval)	Ag-RDT (95% Confidence Interval)	RT-PCR (95% Confidence Interval)	Ag-RDT (95% Confidence Interval)
**-4**	19.05 (11.25-30.41)	0.00 (0.00-5.58)	44.83 (28.41-62.45)	16.67 (7.34-33.56)
**-2**	50.00 (39.88-60.12)	23.60 (15.98-33.39)	48.78 (34.25-63.52)	29.55 (18.16-44.22)
**0**	81.11 (71.82-87.86)	63.27 (53.39-72.14)	85.00 (70.93-92.94)	61.70 (47.43-74.21)
**2**	93.67 (86.03-97.27)	75.00 (65.04-82.87)	85.71 (70.62-93.74)	62.50 (47.03-75.78)
**4**	84.51 (74.35-91.12)	68.75 (57.93-77.85)	77.78 (59.24-89.39)	55.88 (39.45-71.12)
**6**	80.77 (68.10-89.20)	50.00 (38.44-61.56)	73.33 (48.05-89.10)	50.00 (31.43-68.57)
**Cumulative to DPSO 4**	97.98 (92.93-99.44)	86.87 (78.82-92.16)	97.87 (88.89-99.89)	80.85 (67.46-89.58)
**Cumulative to DPSO 6**	100.00 (96.26-100.00)	88.89 (81.19-93.68)	97.87 (88.89-99.89)	82.98 (69.86-91.11)

Table S5: Percent Positivity of Ag-RDT and RT-PCR by Day Past Symptom Onset Among Males and Females among Symptomatic RT-PCR Positive Participants

	**Males**		**Females**	
**Day Past Symptom Onset (DPSO)**	RT-PCR (95% Confidence Interval)	Ag-RDT (95% Confidence Interval)	RT-PCR (95% Confidence Interval)	Ag-RDT (95% Confidence Interval)
**-4**	35.48 (21.12-53.05)	9.38 (3.24-24.22)	22.81 (13.84-35.21)	3.39 (0.93-11.54)
**-2**	48.84 (34.62-63.25)	30.23 (18.60-45.11)	51.19 (40.69-61.59)	23.53 (15.78-33.57)
**0**	82.93 (68.74-91.47)	63.83 (49.54-76.03)	82.14 (72.61-88.87)	63.44 (53.30-72.51)
**2**	91.89 (78.70-97.20)	74.42 (59.76-85.07)	90.54 (81.74-95.34)	70.37 (59.69-79.21)
**4**	87.50 (71.93-95.03)	63.89 (47.58-77.52)	80.65 (69.15-88.57)	64.86 (53.50-74.76)
**6**	77.27 (56.56-89.88)	51.52 (35.22-67.50)	80.49 (65.99-89.77)	47.27 (34.69-60.21)
**Cumulative to DPSO 4**	100.00 (92.44-100.00)	87.23 (74.83-94.02)	96.81 (91.03-98.91)	84.04 (75.33-90.08)
**Cumulative to DPSO 6**	100.00 (92.44-100.00)	91.49 (80.07-96.64)	98.94 (94.22-99.95)	85.11 (76.54-90.92)

Table S6: Percent Positivity of Ag-RDT and RT-PCR by Day Past Exposure among Vaccinated and Unvaccinated RT-PCR Positive Participants

	**Vaccinated Individuals**		**Unvaccinated Individuals**		**All Individuals**	
**Day Past Exposure (DPE)**	RT-PCR	Ag-RDT	RT-PCR	Ag-RDT	RT-PCR	Ag-RDT
**0**	15.79 (5.52-37.57)	5.26 (0.27-24.64)	62.50 (30.57-86.32)	12.50 (0.64-47.09)	29.63 (15.85-48.48)	7.41 (2.06-23.37)
**2**	68.00 (48.41-82.79)	40.74 (24.51-59.27)	78.57 (52.41-92.43)	40.00 (19.82-64.25)	71.79 (56.22-83.46)	40.48 (27.04-55.51)
**4**	87.88 (72.67-95.18)	81.25 (64.69-91.11)	87.50 (63.98-96.50)	81.25 (56.99-93.41)	87.76 (75.76-94.27)	81.25 (68.06-89.81)
**6**	90.91 (76.43-96.86)	85.00 (70.93-92.94)	93.75 (71.67-99.68)	88.89 (67.20-96.90)	91.84 (80.81-96.78)	86.21 (75.07-92.84)
**8**	96.55 (82.82-99.82)	81.82 (65.61-91.39)	78.57 (52.41-92.43)	57.14 (32.59-78.62)	90.70 (78.40-96.32)	74.47 (60.49-84.75)
**10**	96.15 (81.11-99.80)	61.54 (42.53-77.57)	64.29 (38.76-83.66)	14.29 (4.01-39.94)	85.00 (70.93-92.94)	45.00 (30.71-60.17)
**Cumulative to DPE 4**	87.50 (73.89-94.54)	73.68 (57.99-85.03)	90.91 (72.19-97.47)	81.48 (63.30-91.82)	89.04 (79.84-94.34)	77.46 (66.48-85.63)
**Cumulative to DPE 6**	97.92 (89.10-99.89)	93.75 (83.16-97.85)	95.45 (78.20-99.77)	95.45 (78.20-99.77)	97.59 (91.63-99.34)	93.98 (86.66-97.40)

Table S7: Performance of Ag-RDT and RT-PCR by Day Past Exposure among Symptomatic and Asymptomatic RT-PCR Positive Participants

	**Symptomatic Individuals**		**Asymptomatic Individuals**	
**Day Past Exposure (DPE)**	RT-PCR (95% Confidence Interval)	Ag-RDT (95% Confidence Interval)	RT-PCR (95% Confidence Interval)	Ag-RDT (95% Confidence Interval)
**0**	60.00 (23.07-88.24)	0.00 (0.00-43.45)	18.75 (6.59-43.01)	6.67 (0.34-29.82)
**2**	94.12 (73.02-99.70)	55.56 (33.72-75.44)	42.86 (21.38-67.41)	13.33 (3.74-37.88)
**4**	93.55 (79.28-98.21)	90.32 (75.10-96.65)	66.67 (35.42-87.94)	37.50 (13.68-69.43)
**6**	92.11 (79.20-97.28)	88.10 (75.00-94.81)	100.00 (56.55-100.00)	100.00 (60.97-100.00)
**8**	92.59 (76.63-97.94)	83.33 (66.44-92.66)	83.33 (55.20-95.30)	61.54 (35.52-82.29)
**10**	90.00 (69.90-97.21)	47.37 (27.33-68.29)	78.57 (52.41-92.43)	50.00 (28.00-72.00)
